# Unresponsiveness to Chiari Malformation Type I Surgery Can Be Related to the Accompanying Chiari Network

**DOI:** 10.7759/cureus.60896

**Published:** 2024-05-23

**Authors:** Nese Keser, Muhammed B Omar, Isil Kalyoncu Aslan, Ipek Bodur, Bulent T Demirgil

**Affiliations:** 1 Department of Neurosurgery, University of Health Sciences, Medical Faculty of Hamidiye, Bakirkoy Prof. Dr. Mazhar Osman Training and Research Hospital for Psychiatric and Neurological Diseases, Istanbul, TUR; 2 Department of Cardiology, University of Health Sciences, Umraniye Training and Research Hospital, Istanbul, TUR; 3 Department of Neurology, University of Health Sciences, Fatih Sultan Mehmet Training and Research Hospital, Istanbul, TUR

**Keywords:** patent foramen ovale, transthoracic echocardiography (tte), transesophageal echocardiography, right-to-left shunt, paradoxical cerebral embolism, persistent dizziness, unresponsiveness to chiari 1 malformation surgery, transient ischemic attacks, chiari network, chiari 1 malformation

## Abstract

Surgical treatment is indicated for Chiari malformation type 1 (CMI) with tonsillar descent (TD) of >5 mm and other clinical manifestations. However, some patients remain unresponsive to surgery; this is an active topic of discussion. A patient presented to the emergency department with dizziness and an impaired gait. He had a history of hypertension. Magnetic resonance investigations revealed a 9-mm TD and cervical syringomyelia. There was no evidence of interatrial septum pathology on transthoracic echocardiography performed preoperatively. Although his complaints were attributed to CMI and surgery was performed, his symptoms remained persistent. Two years later, when the patient’s dizziness increased, a posterior fossa transient ischemic attack (TIA) was suspected. A large patent foramen ovale (PFO) and Chiari network (CN) were also detected on transesophageal echocardiography. His complaints were resolved following PFO closure. Our case suggests that neurosurgeons should be informed about the results of the companionship of a PFO and CN. Before deciding on CMI surgery for patients with only dizziness complaints, a detailed investigation of accompanying cardiac pathologies is paramount to ensure accurate diagnosis and treatment.

## Introduction

Chiari malformations are a set of anomalies that involve the cerebellum, brainstem, and cervicomedullary junction, with a different degree of downward displacement of cerebellar structures and possible cervical syringomyelia. Chiari malformations were classified by an Austrian pathologist, Hans Chiari, in 1891 into four groups [1]. Chiari malformation type 1 (CMI) is characterized by a cerebellar tonsillar descent (TD) >3-5 mm caudal to the foramen magnum [2]. Surgical treatment is indicated for CMI with a TD >5 mm and clinical manifestations [3]. However, some patients with CMI do not respond to surgery, with the etiopathogenesis and cause for unresponsiveness to surgical treatment remaining unclear and under discussion [3-6].

A patent foramen ovale (PFO) is an anatomical anomaly found in approximately 25% of the general population. Additionally, it is a potential risk factor for paradoxical embolism (PDE) and the leading cause of right-to-left shunt (RLS) [7,8]. However, a small-sized PFO is generally not associated with clinical manifestations [9]. Hans Chiari, who first described CMI, was also the first to report a Chiari network (CN) in the heart’s right atrium in 1897 [10,11]. The CN, typically located at the inferior vena cava-right atrium junction, presents as a reticulated structure extending from the Eustachian valve with multiple threads attached to different right atrium sites, such as the coronary sinus ostium, atrial septum, and terminal crest [11,12]. A CN is identified in 1.3-4% of autopsies. In most cases, as a single presentation, it is not associated with clinical symptoms [12]. However, 83% of this network is associated with PFO [11]. Therefore, it plays an important role in cerebrovascular events via paradoxical embolism (PDE) [11,13,14]. In this report, we present the first CMI case in the literature that did not respond to CMI surgery due to the underlying companionship of PFO and CN.

## Case presentation

**Figure 1:**
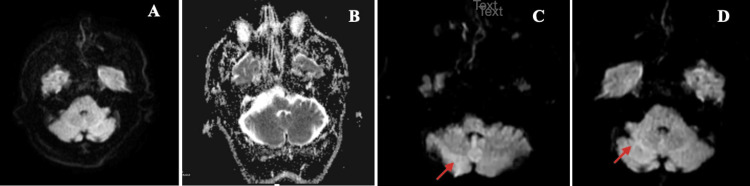
The diffusion-weighted images of the patient Axial diffusion-weighted MRIs of the cerebellum show no restricted diffusion (A, B); Axial diffusion-weighted MRIs of the right cerebellum show restricted diffusion (red arrows) (C, D).

A 45-year-old man presented to the emergency department with acute dizziness and an impaired gait; the patient had a history of hypertension. Examination revealed horizontal nystagmus and ataxic gait, and the patient was admitted to the neurology department. A cranial magnetic resonance (MR) examination revealed a 9-mm TD; the findings of diffusion sequences were unremarkable (Figure 1A, 1B). There was no evidence of interatrial septum pathology on transthoracic echocardiography (TTE). A transient ischemic attack (TIA) was diagnosed, and the patient’s symptoms improved after treatment. Antiaggregant therapy was recommended with follow-up at the neurosurgery outpatient clinic for CMI. The patient was non-compliant with the recommendations at the time.

**Figure 2:**
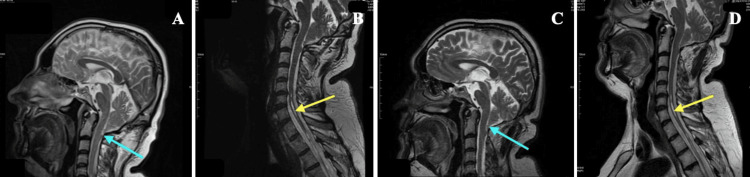
The MRIs of the patient Preoperative sagittal T2-weighted MRI of the brain shows 9-mm tonsillar descending below the foramen magnum (cyan arrow) (A); Preoperative sagittal T2-weighted  MRI of the cervical spine shows cervical syringomyelia (yellow arrow) (B); Sagittal T2-weighted MRI of the brain at second postoperative year (cyan arrow: showing tonsillar descending below the foramen magnum) (C);  Sagittal T2-weighted  MRI of the cervical spine at second postoperative year shows no changes in cervical syringomyelia (yellow arrow) (D).

Eight years later, he was admitted to the neurosurgery outpatient clinic with chronic dizziness and impaired gait complaints. Neurological examination revealed increased deep tendon reflexes of the lower extremities and disturbances in tandem gait. Again, cranial and cervical MR examinations revealed a 9-mm TD and cervical syringomyelia (Figure 2A, 2B). Clinical symptoms were attributed to CMI, and surgery was recommended. A 3x3 cm suboccipital craniectomy and C1 posterior laminectomy were performed. Removal of the atlanto-occipital ligament, dural bands, and adhesions, which were observed to be denser at the foramen magnum level, were performed. The intraoperative and early postoperative periods were without any complications. His imbalance complaints did not wholly resolve even one year after surgery. Deep tendon reflexes of the lower extremities were normal; however, the impairment in tandem gait persisted.

**Figure 3:**
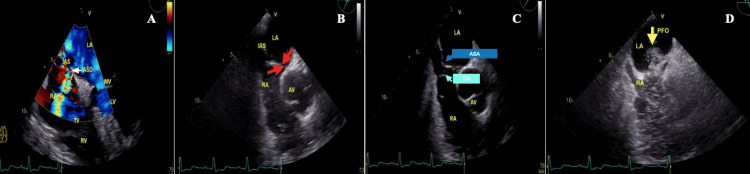
The echocardiographic images of the patient Fourth-degree four-space image, color Doppler shows a transition from LA to RA. Black arrow with a yellow frame, atrial septal defect (ASD) (A); 60-degree aortic valve level. Black arrow with a yellow frame, patent foramen ovale (PFO) (B); aortic valve level. Black arrow with a yellow frame, atrial septal aneurysm (ASA) and Chiari network (CN) (C). Black arrow with a yellow frame, a transition of contrast from the right atrium to the left atrium via PFO (D). RA, right atrium; TV, tricuspid valve; RV, right ventricle; LA, left atrium; MV, mitral valve; LV, left ventricle; IAS, interatrial septum; AV, aortic valve

Furthermore, the MR images did not show observable changes (Figure 2C, 2D). Two years after surgery, the patient presented to the emergency department with increased dizziness. His examination revealed bilateral nystagmus, flattened nasolabial fold on the right side, dysarthria, and mild paresis in the left upper extremity. Since an MRI detected an infarct in the right cerebellar peduncle (Figure 1C, 1D), he was hospitalized at the neurology clinic. A contrast TTE (cTTE), using agitated saline, revealed a PFO and an atrial septal aneurysm (ASA). On TEE, it was observed that PFO and ASA were accompanied by a secundum-type atrial septal defect and a CN (Figure 3). In addition, the diameter of the PFO tunnel was 0.6 cm.

Since a TTE may be sufficient to detect a PFO, to date, contrast TTE (cTTE) is the primary method for PFO identification as it can be overlooked during TTE due to poor imaging quality [15,16]. When a PFO is detected on cTTE, transesophageal echocardiography (TEE) should be performed because TEE enables the detection of PFO with a higher degree of sensitivity [17]. However, it is performed in the second stage, which is semi-invasive [15,16]. In the presence of a large-sized PFO in TEE, closure should be prioritized to reduce the possibility of stroke [18]. Moreover, Alsabbagh et al.'s meta-analysis reported that CN is a high-risk feature in stroke-related PFO and that these cases should be evaluated for PFO closure [19]. Thus, the findings on TEE indicated closure of the PFO, and after PFO closure, the patient’s imbalance complaints gradually improved during the subacute period.

## Discussion

Indications for surgical intervention should only include patients with apparent symptoms attributable to CMI or spinal syrinx. These populations include patients with classic Valsalva-induced headaches, an associated syrinx, and neurological sequelae associated with pathologies such as the foramen magnum, cervicomedullary junction, or lower cranial nerve dysfunction. Peg-like or pointed tonsils and effacement of cerebrospinal fluid (CSF) spaces on MRI are more likely to be associated with symptomatic patients [2]. Since peg-like tonsils and accompanying syringomyelia were detected in the MRI examinations, these findings suggested that the patient's symptoms were associated with these MRI findings. However, most of the CMI is asymptomatic and incidental. Incidental CMI is found in 0.9% of the general adult population [2]. The lack of response to surgical treatment in CMI may be due to the applied technique and errors in case selection. The patient is an example of an appropriate but unnecessary surgical intervention for CMI with an accompanying incidental anomaly. In our case, the lack of response to surgery was not the technique applied but the fact that CMI was encountered incidentally and accompanied by posterior fossa TIAs.

The clinical manifestations of a PFO include cryptogenic stroke, decompression syndrome, platypnea-orthodeoxia syndrome, and peripheral embolism [9,13]. In at least 42% of cerebellar ischemia, the embolism is attributed to a cardiac source such as a PFO or rheumatic heart disease. The most commonly affected areas are those supplied by the posterior inferior cerebellar artery (PICA) and the superior cerebellar artery [7]. The predilection for posterior circulation involvement in PDE has been attributed to microemboli or tiny air bubbles that pass through the atrial defect and enter the vertebral arteries (VAs) more easily than the common carotid arteries [17].

On TEE, a large-sized PFO was accompanied by ASA, a secundum-type atrial septal defect, and a CN. A small-sized PFO is generally not associated with any clinical manifestations. However, an increase in PFO diameter significantly increases the risk of RLS [9]. Although a PFO gradually decreases with age, older patients with a PFO are more susceptible to PDE owing to an increase in the diameter of the PFO with age [13]. An ASA accompanies approximately 35% of patients with a PFO and is associated with large-sized PFOs. Therefore, the risk of PDE also increases [20]. Furthermore, the CN frequently coexists with a PFO (83%) and an ASA (20%); therefore, it plays an important role in cerebrovascular events via PDE [11,13,14]. As a result, all these risk factors for PDE were overlooked in TTE, and posterior circulation TIA symptoms developed in our case.

## Conclusions

In conclusion, our case suggested that investigating radiological findings such as tonsil shape, descending degree, presence or absence of syrinx, and deciding on the appropriate surgical technique is not enough before CMI surgery. Proper patient selection for CMI surgery is more important than the abovementioned criteria for better surgical outcomes. Therefore, neurosurgeons should know the importance of a PFO's and CN's companionship. A thorough cardiac investigation should be performed for CMI patients with only dizziness complaints before deciding on CMI surgery.
